# Gomisin E attenuates pentobarbital-induced sleep deficits in aging-related insomnia: an *in vivo* and *in silico* study implicating cholinergic signaling

**DOI:** 10.3389/fpsyg.2026.1821889

**Published:** 2026-05-14

**Authors:** Fan Yang, Fen Jiang, Xuewei Li

**Affiliations:** 1 Department of Neurology, The First Affiliated Hospital, Hengyang Medical School, University of South China, Hengyang, Hunan, China; 2 Department of Nephrology, The Third Affiliated Hospital of Southern Medical University, Guangzhou, Guangdong, China

**Keywords:** aging-related insomnia, cholinergic pathway, cognitive dysfunction, gomisin E, inflammatory response

## Abstract

**Objective:**

To investigate the therapeutic effects of gomisin E on insomnia in aged rats and to elucidate the underlying mechanisms.

**Methods:**

An aging-related insomnia rat model was established by intraperitoneal injection of D-galactose combined with p-chlorophenylalanine (PCPA). Rats were treated with gomisin E at different doses (5, 15, and 45 mg/kg). Sleep quality, depressive-like behaviors, and cognitive function were evaluated using the pentobarbital sodium-induced sleep test, sucrose preference test, and Morris water maze test, respectively. Potential targets and signaling pathways were screened using bioinformatics analysis. The contents of acetylcholine (ACh) and choline acetyltransferase (ChAT) and acetylcholinesterase (AChE) activities in the hippocampus and ileum were determined using commercial assay kits. Protein levels of muscarinic acetylcholine receptor M1 and M2 (CHRM1 and CHRM2) in the hippocampus were detected by Western blot analysis.

**Results:**

Gomisin E dose-dependently alleviated body weight loss, sleep disorders, depressive-like behaviors, and cognitive dysfunction in aged rats with insomnia, significantly reducing pro-inflammatory cytokine levels. The potential mechanisms of gomisin E involved circadian rhythm regulation, inflammatory responses, the C-type lectin receptor signaling pathway, and cholinergic synapses. Gomisin E increased ACh content and ChAT activity, decreased AChE activity in the hippocampus and ileum, and upregulated the protein expression of CHRM1 and CHRM2 in the hippocampus.

**Conclusion:**

Gomisin E effectively ameliorates D-galactose/PCPA-induced insomnia and the associated depressive-like behaviors and cognitive dysfunction. The underlying mechanisms may involve suppression of systemic inflammation and restoration of cholinergic system homeostasis.

## 1 Introduction

Insomnia is a prevalent sleep disorder in the aging population, which is a public health concern worldwide (Cohen et al., 2022). Epidemiological studies indicate that approximately 30–50% of older adults report chronic sleep disturbances, with a substantial proportion meeting diagnostic criteria for insomnia disorder. The etiology of insomnia in aging is multifactorial, involving physiological, psychological, and environmental factors. Age-related alterations in circadian rhythm regulation, including reduced amplitude of melatonin secretion and impaired suprachiasmatic nucleus function, as well as dysregulation of neuroendocrine signaling and neurotransmitter homeostasis, substantially increase vulnerability to sleep disturbances (Brewster et al., 2022). In addition, chronic inflammation, oxidative stress, and comorbid medical conditions further contribute to the pathogenesis of insomnia in elderly individuals. Importantly, insomnia is not merely a nocturnal complaint but is associated with significant daytime dysfunction and adverse health outcomes. Persistent sleep disturbances have been strongly linked to neuropsychiatric comorbidities, including depression-like behaviors, anxiety, and progressive cognitive decline (Irwin et al., 2022). Consequently, insomnia in the aging population constitutes a major health problem due to its high prevalence, impact on quality of life, and increased healthcare burden.

At the molecular level, the cholinergic system plays a pivotal role in sleep–wake regulation, emotional behavior, and cognitive processing (Bertrand and Wallace, 2020). Central cholinergic neurotransmission, especially in the hippocampus and basal forebrain, is essential for rapid eye movement sleep regulation, memory consolidation, and mood stabilization (Cykowski and Masdeu, 2025). In insomnia, pathological overactivity of cholinergic signaling, particularly via muscarinic M1/M3 receptors and nicotinic α4β2 receptors, leads to hyperarousal and disrupted sleep architecture (Ye et al., 2024). This imbalance, where cholinergic tone fails to decline appropriately during non-REM sleep, represents a key molecular mechanism perpetuating sleep instability.

Current pharmacological interventions for insomnia primarily focus on symptomatic sedation and include benzodiazepines, Z-drugs, and orexin receptor antagonists. However, these agents carry significant risks of tolerance, dependence, next-day sedation, cognitive impairment, and falls, particularly in aged populations (Abad and Guilleminault, 2018). These limitations underscore the urgent need for safer agents that can improve sleep quality while alleviating associated emotional and cognitive impairments.

Gomisin E is a bioactive lignan isolated from *Schisandra chinensis*, a traditional medicinal herb (Wang et al., 2024). Previous studies have demonstrated that *S. chinensis* extracts exert beneficial effects in models of neuroinflammation and oxidative stress (Yan et al., 2021). *S. chinensis* extracts have been shown to inhibit acetylcholinesterase (AChE) activity *in vitro* and *in vivo*, suggesting modulation of cholinergic neurotransmission relevant to cognitive and sleep-related functions (Song et al., 2020). Nevertheless, the potential role of gomisin E in aging-related insomnia and the accompanying behavioral and cognitive abnormalities has not yet been systematically investigated, and whether cholinergic pathway modulation contributes to its therapeutic effects remains unknown.

Based on these observations, we hypothesized that gomisin E ameliorates aging-related insomnia and its associated depression-like behaviors and cognitive impairment by restoring cholinergic system homeostasis and suppressing inflammation. In the present study, we employed a D-galactose and p-chlorophenylalanine (PCPA)–induced aging-related insomnia rat model to systematically evaluate the effects of gomisin E on sleep behavior, emotional state, cognitive performance, inflammatory responses, and cholinergic pathway function. This study innovatively elucidates the role of gomisin E in aging-related insomnia and to link its therapeutic effects to cholinergic regulation. Our findings may provide novel mechanistic insights and suggest gomisin E as a promising candidate for the development of safer and more comprehensive interventions for insomnia in the aging population.

## 2 Materials and methods

### 2.1 Materials

#### 2.1.1 Experimental animal

Two-month-old specific pathogen-free (SPF) male Sprague–Dawley rats (SD rats; weighing 200 ± 20 g; obtained from SJA Laboratory Animal, Changsha, China with license No. SCXK [Xiang] 2021–0002) were housed at temperature 22 ± 2 °C, with relative humidity 50 ± 10%, a 12 h:12 h light:dark cycle, and free access to food and water. Bedding was changed twice weekly. To minimize stress and ensure physiological stability, all rats were acclimatized for 7 days before experiments. All procedures were approved by the Animal Ethics Committee of The First Affiliated Hospital, Hengyang Medical School, University of South China (Approval No.: 2025220422002). Animal experiments were carried out according to the Regulations for the Administration of Experimental Animals and relevant animal welfare guidelines.

#### 2.1.2 Reagents and chemicals

Gomisin E (CAS No. 72960-21-5; purity 96.5%) was purchased from HEPENG Biological (Shanghai, China). D-galactose (IG0541) and PCPA(C8655) were obtained from Solarbio Company (Beijing, China) and Sigma Company (MO, USA), respectively. ELISA kits for rat IL-1β (ml037361), IL-6 (ml064292), and TNF-α (ml002859) and assay kits for ACh (A105), ChAT (A079), and AChE (H529) were purchased from Shanghai Enzyme-linked Biotechnology (Shanghai, China) and Nanjing Jiancheng Bioengineering Institute (Nanjing, China), respectively. Primary antibodies against CHRM1 (A1598), CHRM2 (DF4908) and GAPDH (A19056) were obtained from Abclonal, Affinity and Abclonal.

### 2.2 Methods

#### 2.2.1 Identification of gomisin E and insomnia-related target genes

The simplified molecular-input line-entry system (SMILES) of gomisin E was obtained from the PubChem database, and potential target genes were predicted using the SwissTargetPrediction database (http://www.swisstargetprediction.ch/). Transcriptomic dataset GSE208668, comprising 17 elderly patients with insomnia and 25 elderly controls without insomnia, was downloaded from the GEO database. Differential expression analysis between insomnia and non-insomnia groups was performed using the limma package in R. Genes with |log_2_ fold change| > 1 and adjusted *p* value < 0.05 were considered differentially expressed genes (DEGs). The intersection between gomisin E–related targets and insomnia-associated DEGs was identified using the jvenn online tool.

#### 2.2.2 GO and KEGG enrichment analyses

GO functional annotation and KEGG pathway enrichment analyses of the intersecting genes were conducted using the clusterProfiler R package. The enrichplot R package was used for visualization, and the top 10 most significantly enriched Gene Ontology terms and pathways were presented.

#### 2.2.3 Animal grouping and model establishment

**Figure 1:**
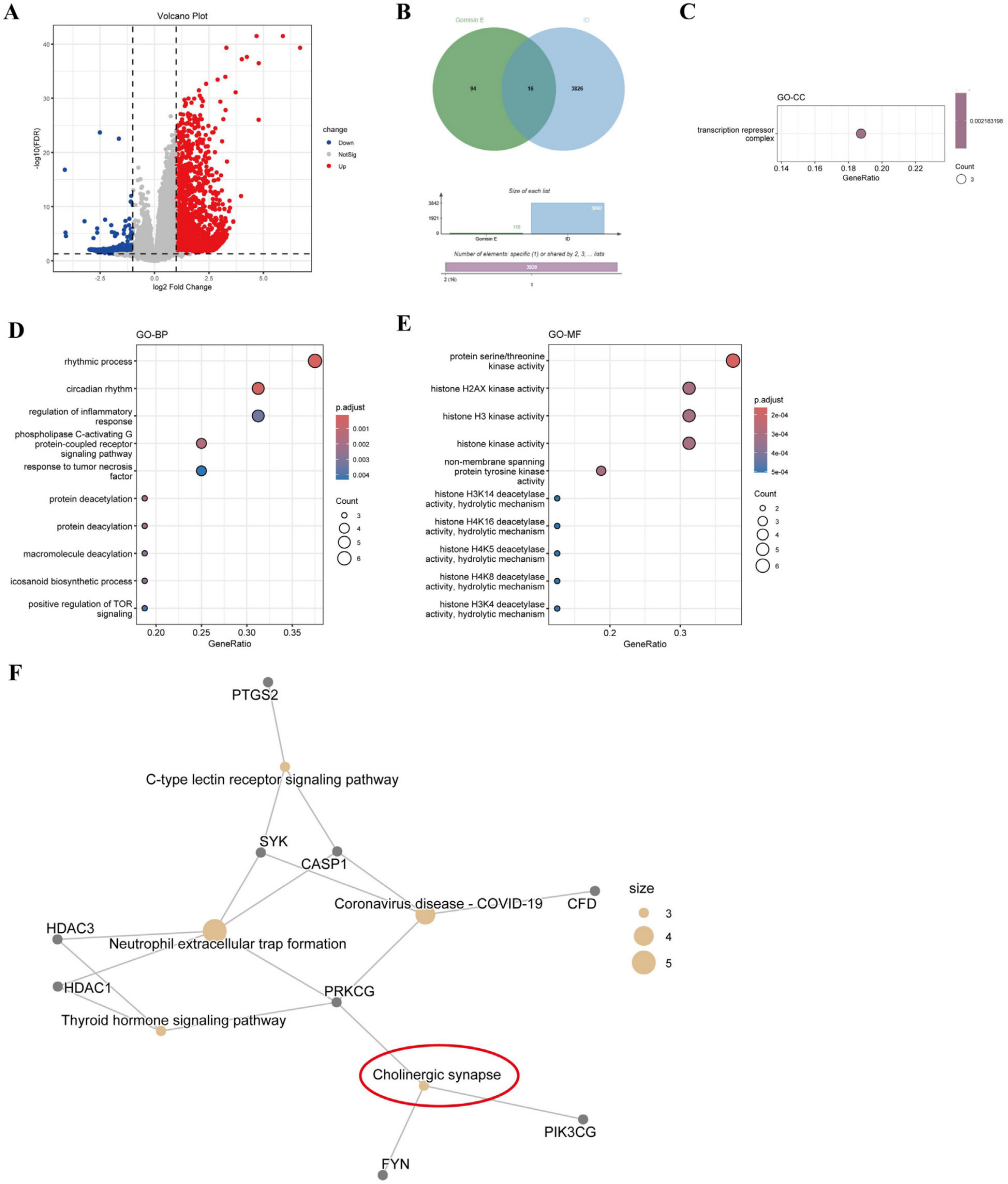
Screening of candidate targets of gomisin E and functional enrichment analysis. **(A)** Volcano plot of DEGs in the GSE208668 dataset. **(B)** Venn diagram showing the intersection between potential targets of gomisin E and insomnia-related differentially expressed genes. **(C–E)** GO enrichment analysis of intersecting genes, including cellular component **(C)**, biological process **(D)**, and molecular function **(E)**. **(F)** KEGG pathway network analysis illustrating the major signaling pathways associated with the intersecting genes.

Rats were randomly divided into five groups (*n* = 8 per group): normal control (NC), Model, Model + GNE_L, Model + GNE_M, and Model + GNE_H. Based on previously reported methods, an aging-related insomnia model was established using D-galactose combined with PCPA (Ren et al., 2020a,b). Briefly, rats in the Model group received subcutaneous injections of D-galactose (120 mg/kg) for 42 consecutive days, followed by intraperitoneal injections of PCPA (300 mg/kg) for 3 days. Rats in the NC group received equal volumes of normal saline. Gomisin E (CAS No. 72960-21-5; purity 96.5%; C_28_H_34_O_9_) was purchased from HEPENG Biological (Shanghai, China). After model establishment, rats were administered normal saline or gomisin E (5, 15, or 45 mg/kg; low, medium, and high doses, respectively) by oral gavage once daily for 7 consecutive days. Dose selection and treatment duration were based on previous studies (Zhang et al., 2014; Ren et al., 2020a). After treatment, body weight was recorded and behavioral tests were performed. Finally, rats were anesthetized, with blood samples collected via the orbital sinus, and rats were euthanized. Brain tissues were harvested and stored at −80 °C for further analysis (Figure 1A).

#### 2.2.4 Pentobarbital-induced sleep test (PIST)

The PIST is used to evaluate hypnotic and sedative effects, which are commonly applied indicators in insomnia models (Abdolmaleki et al., 2016; Ren et al., 2020a). Twenty-four hours after the final treatment, rats were intraperitoneally injected with pentobarbital sodium (35 mg/kg). Sleep onset was defined as the loss of the righting reflex for more than 1 min. The interval from drug administration to disappearance of the righting reflex was measured as sleep onset latency, and the overall duration of the sleep state was subsequently recorded.

#### 2.2.5 Sucrose preference test (SPT)

The SPT was used to assess insomnia-associated depressive-like behavior (Li et al., 2025; Zhou et al., 2025). Prior to testing, rats were singly housed and habituated for 48 h with two bottles containing 1% (w/v) sucrose solution and pure water, respectively; bottle positions were switched every 12 h to avoid side bias. After habituation, rats were deprived of water for 24 h. During the test phase, rats were allowed free access to one bottle of 1% sucrose solution and one bottle of pure water for 12 h. Sucrose preference was calculated as: sucrose preference (%) = [sucrose intake/total fluid intake] × 100%.

#### 2.2.6 Morris water maze (MWM) test

The MWM test was used to evaluate spatial learning and memory (Guo et al., 2025; Liu et al., 2025). The apparatus consisted of a circular pool (50 cm in height, 150 cm in diameter) filled with opaque water maintained at 22 ± 1 °C. The pool was divided into four quadrants, and a platform was placed 1 cm below the water surface in the third quadrant. Distinct visual cues were positioned around the pool. Rats were trained to locate the hidden platform, and escape latency was recorded. If a rat failed to find the platform within 60 s, it was guided to the platform and allowed to remain there for 15 s. Each rat underwent four trials per day for 5 consecutive days. After training, a probe trial was conducted by removing the platform, and the number of crossings over the former platform location was recorded to assess memory retention.

#### 2.2.7 Enzyme-linked immunosorbent assay (ELISA)

Whole blood samples were placed at room temperature for 1 h without agitation to avoid hemolysis. After clotting, serum was obtained by centrifugating samples at 1,200 × g for 10 min at 4 °C to obtain serum. Levels of IL-1β, IL-6, and TNF-α were measured by corresponding ELISA kits. Absorbance was measured at 450 nm.

#### 2.2.8 Measurement of acetylcholine (Ach) level, choline acetyltransferase (ChAT), and AChE activities

Brain and ileum tissues were homogenized in ice-cold normal saline and centrifuged at 10,000 × g for 10 min at 4 °C to collect supernatants. Total protein concentration was determined using a bicinchoninic acid (BCA) protein assay kit (Nanjing Jiancheng Bioengineering Institute, China). Ach content and the activities of ChAT and AChE were measured using commercial assay kits and detected with a microplate reader (Bio-Rad, CA, USA).

#### 2.2.9 Western blot (WB) analysis

Hippocampal tissues were homogenized in ice-cold radioimmunoprecipitation assay lysis buffer containing protease inhibitors (Beyotime Biotechnology, Shanghai, China). Protein concentration was quantified using the BCA method. Equal amounts of protein (30 μg) were separated by sodium dodecyl sulfate–polyacrylamide gel electrophoresis and transferred onto polyvinylidene difluoride membranes. Membranes were blocked with 5% non-fat milk for 1 h at room temperature and incubated overnight at 4 °C with primary antibodies against muscarinic acetylcholine receptor M1 (CHRM1) and muscarinic acetylcholine receptor M2 (CHRM2). After washing, membranes were incubated with horseradish peroxidase–conjugated secondary antibodies for 1 h at room temperature. Protein bands were visualized using enhanced chemiluminescence and quantified with ImageJ software, with glyceraldehyde-3-phosphate dehydrogenase (GAPDH) used as an internal control.

#### 2.2.10 Statistical analysis

All data are presented as mean ± standard deviation (SD). Statistical analyses and graphing were performed using GraphPad Prism 9.0 software. Differences among multiple groups were analyzed by one-way analysis of variance (ANOVA) followed by Tukey's *post hoc* test. A *p* value < 0.05 was considered statistically significant.

## 3 Results

### 3.1 Potential role and mechanisms of gomisin E in aging-related insomnia by bioinformatics

Gomisin E is a bioactive lignan isolated from *S. chinensis*, a traditional medicinal herb (Wang et al., 2024). To preliminarily explore its potential mechanisms of action in aging-related insomnia, bioinformatics analyses were performed. Differential expression analysis of the GSE208668 dataset (|log2FC| > 1, adj.P.Val < 0.05) identified 3,842 insomnia-related DEGs, including 2,222 upregulated and 1,620 downregulated genes (Figure 1A). A total of 110 potential gomisin E target genes were predicted using the SwissTargetPrediction. After intersecting the predicted targets with the DEGs, 16 candidate target genes were obtained (Figure 1B) for subsequent functional analysis.

GO and KEGG enrichment analyses were performed on the intersecting genes. The results showed that, in terms of biological processes, these genes were mainly enriched in pathways related to circadian rhythm regulation, inflammatory response, and positive regulation of TOR signaling, which are associated with rhythmicity and immune-inflammatory processes (Figure 1C). Regarding CC, they were primarily enriched in the transcription repressor complex (Figure 1D). In MF, genes were enriched in histone deacetylase activity, histone kinase activity, and protein serine/threonine kinase activity (Figure 1E). KEGG network analysis further indicated enrichment in pathways related to C-type lectin receptor signaling, COVID-19, NET formation, cholinergic synapse, and thyroid hormone signaling, all of which are closely associated with sleep regulation, immune responses, and neuroendocrine function (Figure 1F). Collectively, the bioinformatics analysis suggests that gomisin E may be associated with multiple biological processes relevant to sleep disorders, including immune-inflammatory responses, circadian rhythm regulation, cholinergic signaling, and epigenetic modifications.

### 3.2 Gomisin E normalizes body weight and sleep behaviors in D-gal/PCPA–induced aging-related insomnia rats

**Figure 2:**
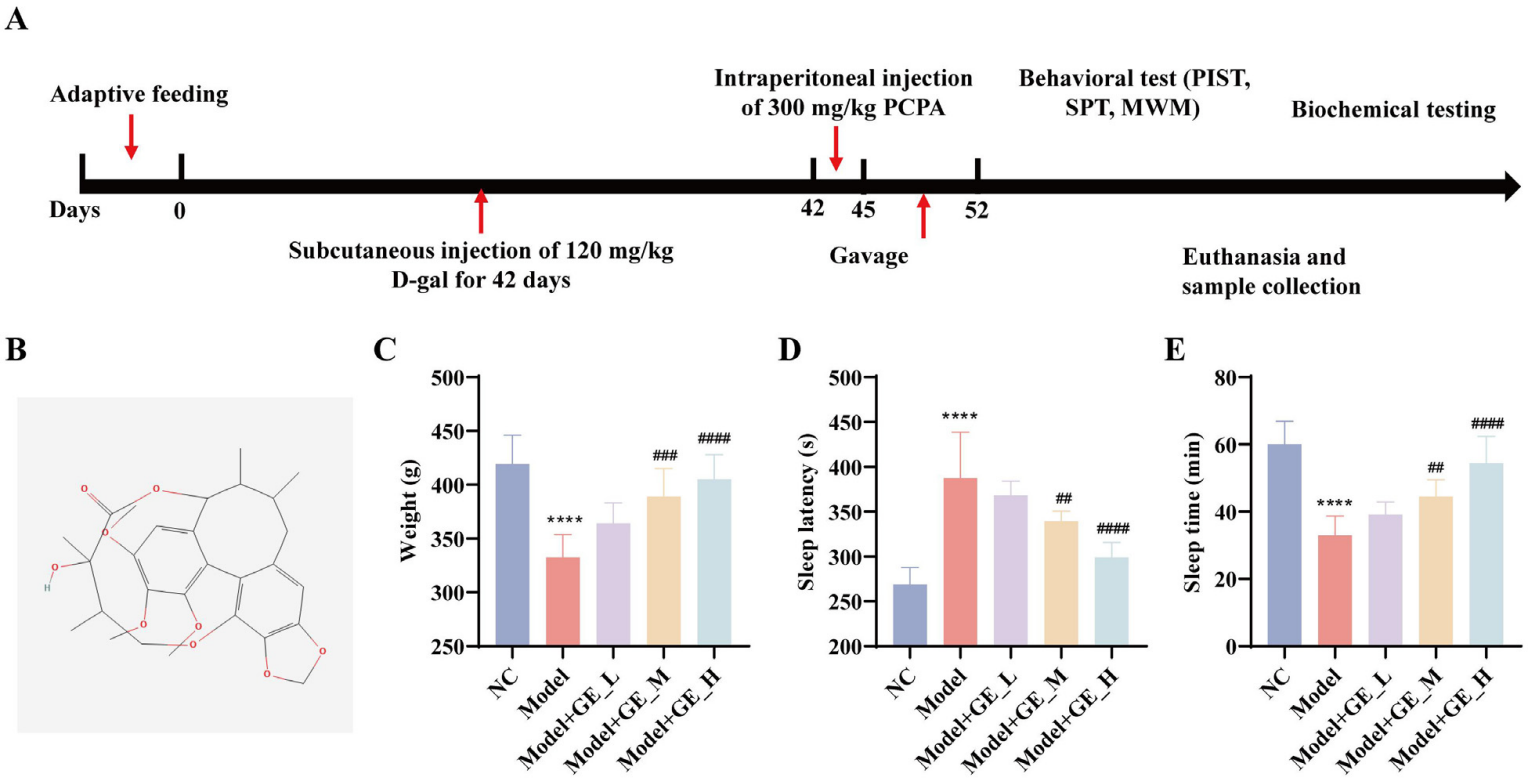
Effects of Gomisin E on body weight and sleep behaviors in aging-related insomnia rats. **(A)** Schematic diagram of the D-gal/PCPA–induced aging-related insomnia model and Gomisin E administration protocol. **(B)** Chemical structure of Gomisin E (PubChem database). **(C)** Changes in body weight among different groups after Gomisin E treatment. **(D, E)** Effects of Gomisin E on pentobarbital sodium–induced sleep, including sleep latency **(D)** and total sleep duration **(E)**. ****p < 0.0001 vs. NC group. ^*##*^*p* < 0.01, ^*###*^*p* < 0.001, ^*####*^*p* < 0.0001 vs. model group.

To evaluate the effects of gomisin E in aging-related insomnia, a D-gal/PCPA–induced accelerated aging insomnia rat model was established, and gomisin E was administered as shown in Figure 2A. The molecular structure of gomisin E is presented in Figure 2B. Body weight analysis revealed that, rats in the Model group exhibited an approximately 20% reduction in body weight, whereas gomisin E treatment significantly restored body weight in a dose-dependent manner (Figure 2C). In the PIST, rats in the NC group showed the shortest sleep latency and the longest total sleep time, while the Model group demonstrated significantly prolonged sleep latency and reduced total sleep duration (*p* < 0.05). Gomisin E administration markedly shortened sleep latency and prolonged total sleep time in a dose-dependent manner (*p* < 0.05; Figures 2D, E). These findings indicate that gomisin E significantly alleviates body weight loss and sleep disturbances induced by D-gal/PCPA.

### 3.3 Gomisin E alleviates depressive-like behaviors and cognitive impairment in D-gal/PCPA–induced aging-related insomnia rats

**Figure 3:**
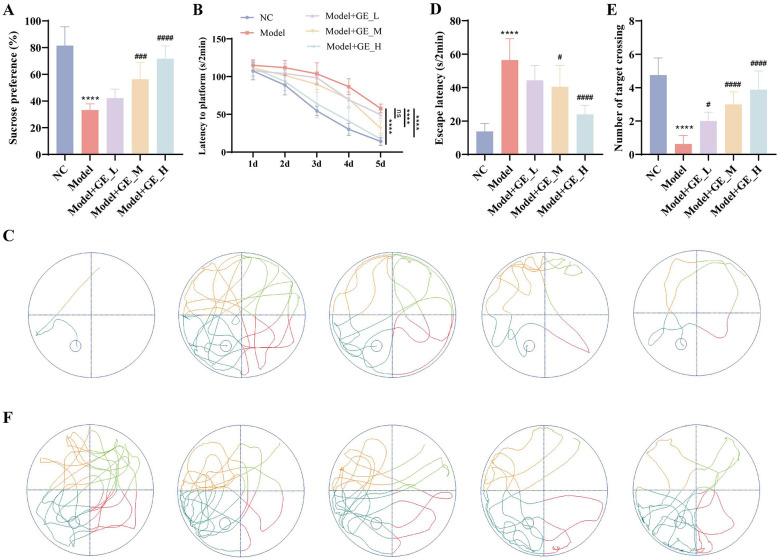
Effects of Gomisin E on depressive-like behaviors and cognitive impairment in D-gal/PCPA–induced aging-related insomnia rats. **(A)** SPT for the assessment of depressive-like behaviors in each group. **(B**, **C)** MWM test evaluating escape latency during the training period (1d−5d). **(D**–**F)** MWM test evaluating the latency and the number of target platform crossings during the experimental period. ****p < 0.0001 vs. NC group. ^#^*p* < 0.05, ^*##*^*p* < 0.01, ^*###*^*p* < 0.001, ^*####*^*p* < 0.0001 vs. model group.

To further assess the effects of gomisin E on behavioral and cognitive dysfunction, SPT and MWM tests were performed. In the SPT, sucrose consumption was reduced in the Model group, indicating pronounced depressive-like behavior (*p* < 0.0001). Gomisin E treatment significantly increased sucrose intake in a dose-dependent manner, with the high-dose group showing the most prominent improvement (Figure 3A).

As training progressed, escape latency decreased in all groups, indicating acquisition of spatial learning. However, the Model group consistently exhibited longer escape latencies than the NC group throughout the training period, suggesting impaired learning ability. From day 2 onward, rats treated with low-, medium-, and high-dose gomisin E showed significantly shorter escape latencies than those in the Model group, with the high-dose group displaying the steepest decline and performance closest to the NC group (Figures 3B, C). In the probe trial, the Model group exhibited significantly fewer platform crossings and reduced time spent in the target quadrant, indicating impaired spatial memory retention. Gomisin E treatment markedly increased the number of platform crossings and prolonged target-quadrant dwell time in a dose-dependent manner, suggesting improved spatial memory performance (*p* < 0.05, Figures 3D–F). Collectively, these results demonstrate that gomisin E effectively ameliorates D-gal/PCPA–induced insomnia and its associated depressive-like behaviors and cognitive deficits, with efficacy increasing with dose.

### 3.4 Gomisin E attenuates inflammatory responses in D-gal/PCPA–induced aging-related insomnia rats

**Figure 4:**
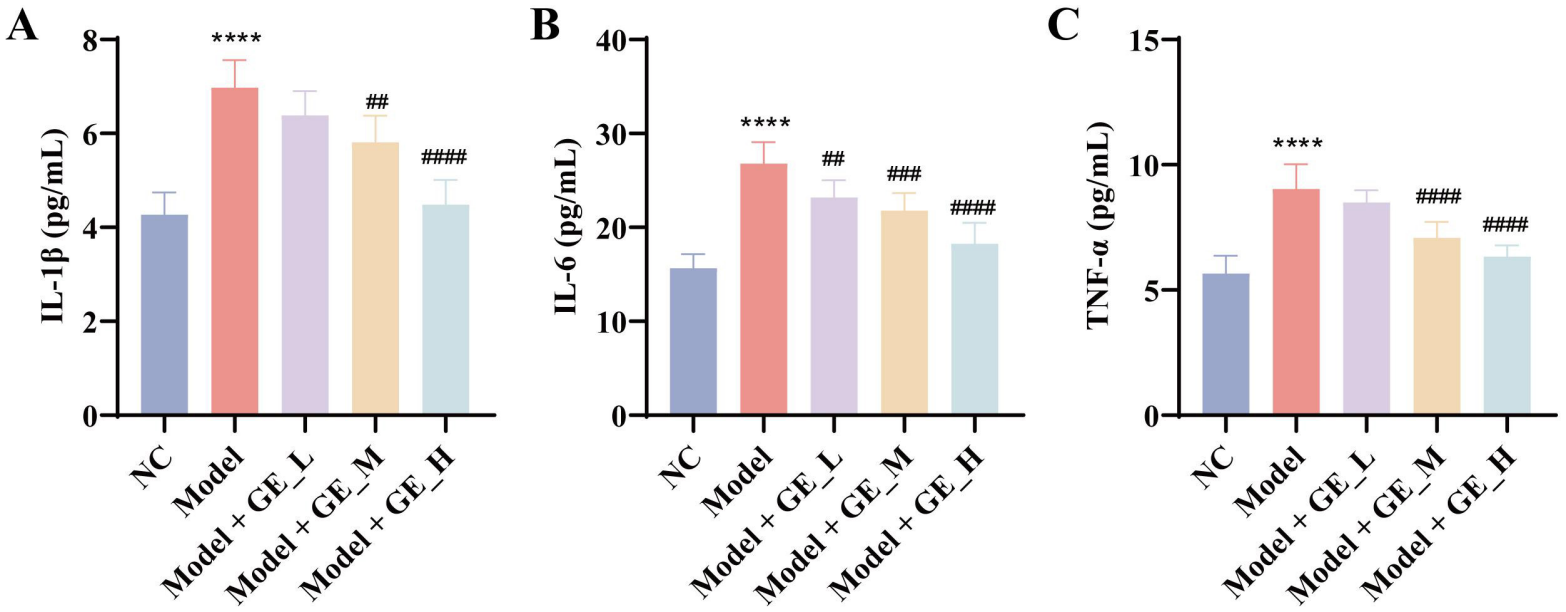
Effects of Gomisin E on inflammatory cytokine levels in D-gal/PCPA–induced aging-related insomnia rats. **(A–C)** Serum levels of IL-1β **(A)**, IL-6 **(B)**, and TNF-α **(C)** measured by ELISA. ****p < 0.0001 vs. NC group. ^*##*^*p* < 0.01, ^*###*^*p* < 0.001, ^*####*^*p* < 0.0001 vs. model group.

To evaluate the anti-inflammatory effects of gomisin E, levels of IL-1β, IL-6, and TNF-α were measured by ELISA. Compared with the NC group, the Model group showed significantly elevated levels of these inflammatory cytokines (*p* < 0.0001), indicating a pronounced systemic inflammatory response induced by D-gal/PCPA. Gomisin E administration significantly reduced serum IL-1β, IL-6, and TNF-α levels, showing a dose-dependent trend, with the high-dose group exhibiting the strongest inhibitory effect (*p* < 0.01; Figures 4A–C). These results suggest that gomisin E effectively alleviates inflammation in aging-related insomnia rats.

### 3.5 Gomisin E restores cholinergic pathway dysfunction in D-gal/PCPA-induced aging-related insomnia rats

**Figure 5:**
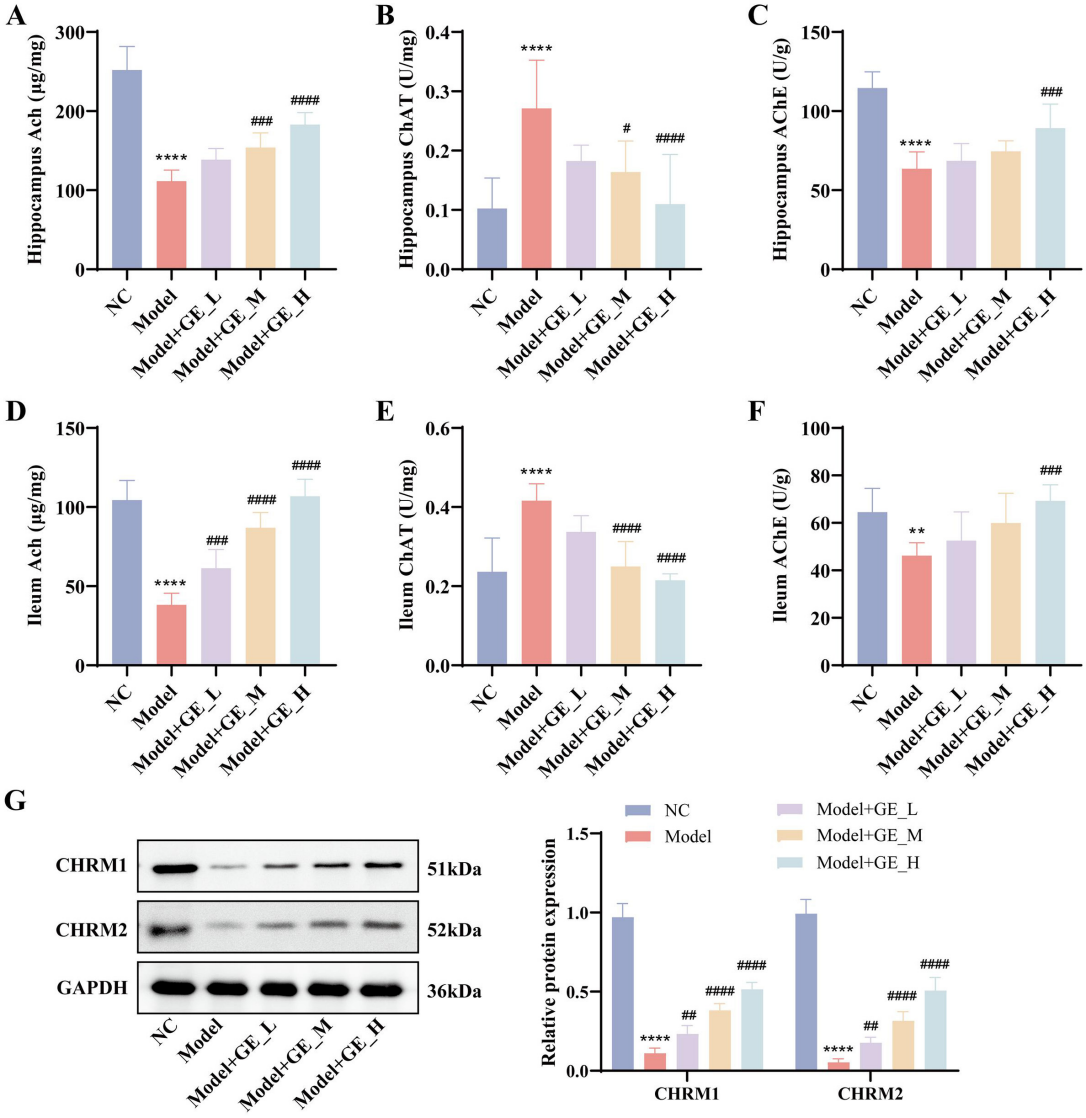
Effects of gomisin E on the cholinergic pathway in D-gal/PCPA–induced aging-related insomnia rats. **(A**–**F)** ACh content and activities of ChAT and AChE in the hippocampus and ileum determined by commercial assay kits. **(G)** Protein expression levels of CHRM1 and CHRM2 in the hippocampus detected by WB and corresponding quantitative analysis. **p < 0.01,****p < 0.0001 vs. NC group. ^#^*p* < 0.05, ^*##*^*p* < 0.01, ^*###*^*p* < 0.001, ^*####*^*p* < 0.0001 vs. model group.

Previous studies have demonstrated that cholinergic neuronal loss and neurotransmitter homeostatic imbalance are critically involved in the development of insomnia (Bah et al., 2010; Ye et al., 2025). Combined with the bioinformatics results, we hypothesized that gomisin E exerts its anti-insomnia effects by modulating the cholinergic system. Therefore, ACh levels and ChAT and AChE activities were measured in the hippocampus and ileum. As shown in Figures 5A–F, compared with the NC group, the Model group exhibited significantly reduced ACh content and ChAT activity, along with markedly increased AChe activity in both tissues (*p* < 0.0001), indicating cholinergic dysfunction induced by D-gal/PCPA. Gomisin E treatment significantly increased ACh levels and ChAT activity while reducing AChE activity in a dose-dependent manner (*p* < 0.05).

Furthermore, WB analysis demonstrated that CHRM1 and CHRM2 protein expression levels in the hippocampus were significantly downregulated in the Model group compared with the NC group (*p* < 0.0001). Gomisin E administration markedly upregulated CHRM1 and CHRM2 expression relative to the Model group in a dose-dependent manner (*p* < 0.01; Figure 5G). Taken together, these results indicate that gomisin E effectively ameliorates D-gal/PCPA-induced aging-related insomnia by restoring cholinergic pathway dysfunction.

## 4 Discussion

Aging-related insomnia is a prevalent and progressively worsening sleep disorder in older populations (Jaqua et al., 2023). Although pharmacological agents such as benzodiazepines and non-benzodiazepine hypnotics are commonly prescribed, their long-term use in the elderly is limited by adverse effects including tolerance, dependence, cognitive impairment, and increased fall risk (Capiau et al., 2023). Consequently, there is a pressing need to identify safer and more effective therapeutic compounds that can simultaneously improve sleep disturbances and mitigate associated neurobehavioral deficits in aging populations. Our findings indicate that gomisin E, a naturally derived lignan, not only ameliorates sleep disturbances but also simultaneously improves depressive-like behaviors, cognitive dysfunction, systemic inflammation, and cholinergic imbalance in an aging-related insomnia model.

Our experimental results showed that gomisin E dose-dependently normalized body weight and improved sleep behaviors, as evidenced by reduced sleep latency and prolonged total sleep time, in D-gal/PCPA-induced aging-related insomnia rats. The improvement in body weight and sleep parameters suggests that gomisin E may alleviate systemic and central dysregulation induced by D-gal/PCPA (Rosenblum et al., 2025). Aging-related insomnia is often accompanied by metabolic imbalance, chronic inflammation, and disrupted neurotransmitter homeostasis, all of which contribute to weight loss and sleep fragmentation (Duan et al., 2023). Gomisin E may counteract these alterations by restoring neurochemical balance and reducing physiological stress, thereby improving sleep efficiency and overall physical condition.

Furthermore, we found that gomisin E dose-dependently alleviated depressive-like behaviors and improved learning and memory deficits in D-gal/PCPA-induced aging-related insomnia rats, while decreasing inflammatory cytokine levels. Consistently, previous studies have demonstrated that Schisandra- derived compounds ameliorate cognitive impairment through antioxidative and anti-inflammatory mechanisms (Xu et al., 2019). However, the present study extends these findings by specifically linking gomisin E to the improvement of behavioral and cognitive dysfunction secondary to aging-related insomnia, a relationship that has been rarely characterized before. Aging-related insomnia is frequently accompanied by affective disturbances and cognitive decline, which are closely associated with neuroinflammation, neurotransmitter imbalance, and hippocampal dysfunction (Furukawa et al., 2021). The observed improvement in sucrose preference suggests that gomisin E may mitigate anhedonia by restoring reward-related neural circuits disrupted by chronic sleep disturbance. Meanwhile, the enhanced spatial learning and memory performance in the MWM indicates a protective effect of gomisin E on hippocampus-dependent cognitive processes. These effects may be mechanistically attributed to the ability of gomisin E to suppress inflammation-induced synaptic dysfunction and to restore neurotransmitter homeostasis (Zhang et al., 2024). Besides, elevated levels of IL-1β, IL-6, and TNF-α have been consistently reported in both clinical insomnia and experimental sleep deprivation models, supporting inflammation as a core pathological feature of sleep disorders (Liu et al., 2024). The present findings add novel evidence that gomisin E effectively suppresses inflammation specifically in the context of aging-related insomnia. Proinflammatory cytokines such as IL-1β, IL-6, and TNF-α can directly disrupt sleep–wake regulation by altering hypothalamic signaling and impairing synaptic plasticity within sleep- and cognition-related brain regions (Rahman et al., 2015). The ability of gomisin E to markedly reduce these cytokines suggests that its therapeutic effects may be mediated, at least in part, through attenuation of inflammation-driven neural dysfunction.

In this study, we selected low (5 mg/kg), medium (15 mg/kg), and high (45 mg/kg) doses of gomisin E for *in vivo* evaluation of its effects on aging-related insomnia in rats. This dosing regimen was designed to span a pharmacologically relevant range that could reveal dose-dependent efficacy while remaining within a range previously used for structurally and functionally related *Schisandra* lignans in preclinical research (Jiang et al., 2024). A key reference informing our dose choices is studies on gomisin N, a major lignan component of *S. chinensis* with similar pharmacological properties. Gomisin N has been shown to produce dose-dependent increases in sleep duration in pentobarbital-treated rodents over a range of 5–45 mg/kg (intraperitoneal) without inducing sedation at the highest dose, indicating this range is biologically active and well tolerated in rodent models of sleep regulation (Zhang et al., 2014). Although direct pharmacokinetic and toxicity data for gomisin E monomer are limited, the broader literature on *Schisandra* lignans suggests that compounds in this class have relatively favorable safety profiles in rodents at comparable doses.

Mechanically, GO and KEGG analyses, together with experimental validation, indicate that gomisin E ameliorates aging-related insomnia by modulating immune-inflammatory pathways and restoring cholinergic signaling, particularly through upregulation of CHRM1 and CHRM2. Previous studies on *S. chinensis* extracts and related lignans have highlighted their anti-inflammatory and neuroprotective properties, as well as their potential to influence cholinergic function (Bah et al., 2010; Ye et al., 2025). Owona et al. have reported that the extract's multimodal modulation of cholinergic, GABAergic, and monoaminergic systems, combined with its antioxidant and anti-inflammatory properties, positions it as a promising candidate for neuroprotection in menopausal women (Owona et al., 2024). Our bioinformatics-driven findings, combined with biochemical and protein expression analyses, therefore extend existing knowledge and provide novel mechanistic insight into the role of gomisin E in sleep regulation. Aging-related insomnia has been increasingly associated with gut-brain axis dysfunction, characterized by intestinal neurotransmitter imbalance, low-grade inflammation, and impaired neural signaling to sleep- and cognition-related brain regions (Wang et al., 2022). The gut–brain axis represents a bidirectional communication network linking the enteric and central nervous systems through neural, endocrine, and immune pathways. In the present study, gomisin E treatment significantly increased ileal ACh levels and ChAT activity while reducing AChE activity, suggesting a net enhancement of enteric cholinergic tone. Given that the vagus nerve serves as a primary conduit for gut-derived signals to reach the brain (Wang et al., 2025), it is plausible that increased ileal ACh acts on vagal afferent fibers, which express nicotinic acetylcholine receptors (nAChRs), particularly the α7 subunit. Activation of vagal afferents by enteric ACh or by ACh released from nearby parasympathetic endings could trigger ascending projections to the nucleus tractus solitarius (NTS) and subsequently to the basal forebrain, hypothalamus, and limbic structures—regions critically involved in sleep–wake regulation, mood, and cognition (Norup Hertel et al., 2024). Supporting this concept, previous studies have shown that peripheral cholinergic stimulation can modulate central inflammation and behavioral states via the cholinergic anti-inflammatory pathway and vagal-dependent mechanisms (Halder and Lal, 2021). In the context of insomnia, which is characterized by central hyperarousal and neuroinflammation, enhanced ileal cholinergic signaling may send counter-regulatory signals to the brain, dampening excessive arousal and restoring sleep architecture. The simultaneous improvement of ileal and hippocampal cholinergic markers observed in this study raises the possibility that gomisin E may exert its anti-insomnia effects, at least in part, through normalization of gut-brain cholinergic communication. Importantly, muscarinic acetylcholine receptors CHRM1 and CHRM2 are key mediators of cholinergic signaling in the hippocampus and play critical roles in arousal regulation, memory consolidation, and rapid eye movement sleep (Fujita et al., 2022). The observed downregulation of CHRM1 and CHRM2 in insomnia rats implies reduced cholinergic receptor responsiveness during aging-related insomnia.

From a clinical translational perspective, gomisin E shows promise as a candidate for aging-related insomnia with comorbid mood and cognitive impairments. Its ability to normalize sleep while concurrently alleviating inflammation and cognitive deficits suggests potential advantages for elderly patients, in whom insomnia rarely occurs in isolation. As a bioactive compound derived from *S. chinensis*, gomisin E may also have favorable safety and tolerability profiles, supporting its future development as a functional drug or adjuvant therapy.

Several limitations of the present study should be acknowledged. First, the current experimental design does not allow us to definitively distinguish whether gomisin E acts preferentially on peripheral vs. central cholinergic systems, and this remains an unresolved question. Second, the absence of a positive control group (e.g., diazepam or zolpidem) limits direct comparison with clinically used hypnotics and weakens translational relevance; future studies will incorporate standard positive controls to better evaluate the relative efficacy of gomisin E. Third, although group sizes (*n* = 8) were determined based on previously published studies in similar animal models (Yu et al., 2022; Kim et al., 2024; Li et al., 2025; Qiu et al., 2026), a formal power analysis was not performed, and this should be addressed in future investigations. Fourth, the 7-day treatment duration is relatively short compared to the overall 42-day aging induction period. However, it should be noted that the D-galactose treatment primarily induces an aging-like phenotype, whereas insomnia is established during the final 3 days via PCPA administration; thus, the intervention mainly targets insomnia-related symptoms rather than reversing the entire aging process. Nevertheless, future studies with extended treatment windows are warranted. Fifth, the D-galactose-induced model represents an accelerated aging phenotype driven largely by oxidative stress and does not fully recapitulate natural aging-related insomnia (e.g., circadian or orexin dysregulation); therefore, our conclusions have been carefully moderated to avoid overgeneralization. Sixth, our results only demonstrate a correlation between cholinergic pathway modulation and sleep improvement; causal relationships require further interventional studies, such as receptor blockade or genetic manipulation. Seventh, peripheral inflammatory cytokines cannot fully represent central neuroinflammation; future work should measure inflammatory markers in specific brain regions to strengthen mechanistic insights. Eighth, while inflammatory cytokines (IL-1β, IL-6, TNF-α) were measured only in peripheral serum, the Discussion extensively attributed therapeutic effects to central neuroinflammation without exploring the relationship between peripheral and central markers—this weakens the credibility of the neuroinflammation mechanism argument and should be rectified in future studies using parallel central and peripheral sampling. Ninth, the bioinformatics analysis was hypothesis-generating rather than experimental validation. The enrichment analysis based on a small gene set (*n* = 16) has potential bias and limited robustness, and species differences between human-derived datasets and our rat model exist. Moreover, transcriptomic profiling in our animal model, which would better fit the mechanistic exploration, was not performed due to limitations in laboratory conditions and research funding. The low intersection rate suggests that gomisin E may act on a subset of relevant targets rather than broadly affecting the entire insomnia-related transcriptome. Future studies should incorporate RNA sequencing from target brain regions in the same animal model and validate candidate targets using molecular approaches such as siRNA knockdown or CRISPR-based editing.

## 5 Conclusion

The present study demonstrates that gomisin E significantly improves sleep quality, attenuates body weight loss, alleviates depressive-like behaviors, and ameliorates impairments in spatial learning and memory in D-galactose/PCPA–induced aging-related insomnia rats, while also mitigating systemic inflammatory responses. The protective effects of gomisin E appear to be mediated, at least in part, through the restoration of functional balance within the cholinergic pathway. These findings provide experimental evidence supporting gomisin E as a potential therapeutic candidate for the treatment of aging-related insomnia and its associated comorbidities.

## Data Availability

The original contributions presented in the study are included in the article/supplementary material, further inquiries can be directed to the corresponding author.
